# Personality and good business judgement: the bright and dark side of business reasoning

**DOI:** 10.3389/fpsyg.2025.1565485

**Published:** 2025-04-28

**Authors:** Adrian Furnham, Ryne A. Sherman

**Affiliations:** ^1^BI Norwegian Business School, Oslo, Norway; ^2^Department of Marketing, BI Norwegian Business School, Oslo, Norway; ^3^Hogan Assessment Systems, Tulsa, OK, United States

**Keywords:** bright side, dark side, personality, business reasoning, bright-side

## Abstract

The current study explored the relationship between measures of “bright-side” and “dark-side” personality traits and business reasoning (BR)/judgment using the Hogan Business Reasoning Inventory (HBRI). Participants were a global sample (*N* = 2,342) who completed the Hogan Personality Inventory (HPI), a bright-side trait measure; the Hogan Development Survey (HDS), a dark-side trait measure; and the HBRI, which is similar to a measure of general cognitive ability. The analyses showed gender effects (men scored higher) but not age effects. Correlation and regression analyses showed that Learning Approach and Adjustment traits were positively associated with business reasoning, while Prudence and Inquisitive traits were negatively associated with business reasoning. In cases where significant dark-side factor relationships were observed, they were negatively associated with business reasoning, except for Reserved and Imaginative traits. However, these traits accounted for relatively little of the variance (approximately 5%) in business reasoning. Stable, ambitious, and intellectually curious individuals who are not high on Conscientiousness and have few dark-side traits appear to be better at business reasoning.

## Introduction

There is a considerable body of literature on the relationship between personality and cognitive ability (Bardach et al., 2023, 2024; Bédard and Le Corff, 2020; Chamorro-Premuzic and Arteche, 2008; DeYoung, 2011; DeYoung et al., 2014; Furnham et al., 2007a; Furnham et al., 2007b; Furnham, 2018a; Furnham, 2018b; Furnham, 2023; George and Zhou, 2001; von Stumm et al., 2011). This study focused on the relationship between business reasoning (BR), assessed through qualitative and quantitative reasoning, and personality. In doing so, we used the terms business reasoning, intelligence, and applied intelligence interchangeably. The study addressed the question of the extent to which one might infer reasoning, using real-world problems in a business context, from personality data. Furthermore, it extended the related literature by examining both bright-and dark-side personality traits and reasoning ability.

Hambrick et al. (2020) argued that “the ability to solve problems is not just an aspect or feature of intelligence—it is the essence of intelligence” (p. 553). Lakin and Kell (2020) argued that reasoning, problem-solving, and decision-making represent different but overlapping aspects of human intelligence. They also pointed out that one of the key controversies regarding reasoning abilities is the extent to which individual differences in reasoning abilities overlap with individual differences in working memory capacity.

There has long been an interest in the relationship between psychometrically assessed personality and intelligence and in how they may have shaped or influenced each other (Anglim et al., 2022; Cuppello et al., 2023a,b; Furnham and Treglown, 2018). In this study, we used two very well-developed and established measures of personality and a relatively new applied measure of cognitive ability. There were many personality measures to choose from, and the majority of studies in this area have used established Big Five measures of bright-side personality (Furnham, 2023). However, we selected a well-established and validated measure specifically designed to assess personality in the workplace (Hogan J. et al., 2007; Hogan R. et al., 2007).

Furthermore, there are almost no measures of dark-side traits apart from the one we used in this study. Bensi et al. (2010) concluded from their highly salient study that personality predicts peculiar ways of reasoning and decision-making, which, in turn, are involved in the formation and/or maintenance of psychological disorders.

There are two major theories in this area linking personality and intelligence. First, compensation theory suggests that Conscientiousness acts as a “coping/reimbursing strategy” for less intelligent, but ambitious and competitive, people in particular settings. Relatively less intelligent individuals may become more methodical, organized, thorough, and persistent (i.e., Conscientious) to compensate for their relative lack of intelligence in a highly competitive educational or work environment. In this sense, intelligence shapes personality by influencing certain personality traits. Therefore, it may be hypothesized that the bright-side trait Prudence and the dark-side trait Diligence would be negatively correlated with business reasoning.

Second, investment theory posits that individual differences in knowledge attainment result from differences in cognitive ability and the propensity to apply and invest that ability. *Investment* personality traits, such as Openness to Experience and Need for Cognition, are related to IQ. It should be noted that other traits have been associated with IQ test performance, such as Anxiety or Neuroticism, as test anxiety reduces test performance (Moutafi et al., 2005). Similarly, it may therefore be hypothesized that the bright-side trait Inquisitive and the dark-side trait Imaginative would be positively correlated with business reasoning.

Reviews suggest that the relationship between personality and intelligence—the two essential poles of differential psychology—is robust but weak, with significant correlations being small in magnitude and effect sizes modest. Undoubtedly, genetics explains the covariance between personality traits and reasoning/IQ. However, a new meta-analysis suggested that the relationship is stable and significant. In a study that summarized 60,690 relations between 79 personality and 97 cognitive ability constructs across 3,543 meta-analyses—based on data from millions of individuals—Stanek and Ones (2023) concluded that the links between personality traits and cognitive ability are not limited to the trait Openness and its components. Some aspects and facets of Neuroticism, Extraversion, and Conscientiousness are also related to primary and specific abilities.

There are also a number of studies on the nature and correlates of business decision-making and competencies (Ayal et al., 2015; Brown and Stuhlmacher, 2020; Doe et al., 2017; Hänninen et al., 2014; Zhang and Highhouse, 2018). Some researchers in this area have been concerned with developing new and robust measures to assess decision-making in a business context (Hamilton et al., 2017; Stanovich, 2016, 2018). Others have been concerned specifically with individual difference correlates of this decision-making (Bruine de Bruin et al., 2007; Dalal and Brooks, 2014). There are also reviews and meta-analyses available on this topic (Phillips et al., 2016; Macke et al., 2022; Zhang and Highhouse, 2018).

The majority of the studies in this topic have used the Big Five personality traits framework to measure personality. The majority of researchers have assumed that both personality and intelligence are relatively stable and unrelated traits (Ones et al., 2024). However, while there are strong correlations between measures of the same/similar personality traits (more so for bright-side than dark-side traits), there is a considerable disagreement about how to define and measure intelligence.

### Ability measures in business settings

It is often asserted that decision-making or reasoning style, rather than the “raw cognitive ability” of a business executive, may be more important to the overall success of any leader in an organization (Sternberg, 1997). Other researchers have argued that business reasoning is simply a manifestation of applied cognitive ability, which can be measured using a standard intelligence test. Certainly, in applied fields, people call for tests of face validity, which partly accounts for the development and use of tests such as the Watson–Glaser test of critical thinking (Watson and Glaser, 1980).

The issue of measuring ability and intelligence with acceptable face validity in both business and educational settings has been recognized for some time (Edwards and Schleicher, 2004). The whole issue of intelligence testing has become more sensitive, mainly due to concerns around group differences, leaving selectors in a dilemma. While they know intelligence is a robust predictor of success at work (Chamorro-Premuzic and Furnham, 2010; Hough et al., 2001), a “solution” is not using an obvious ability or intelligence test but instead measuring it in terms of more face valid measures, such as solving business problems through reasoning.

This study focused on personality correlates of business reasoning using not only a face valid but also construct valid measure of business reasoning. The test is not merely a disguised measure of cognitive ability but assesses rational decision-making in an applied business context.

Hogan et al. (2009) proposed conceptualizing intelligence in terms of capacity for meta-representation: the ability to reflect on one’s performance and identify problems and performance inhibitors. They argued that it was important to measure intelligence in terms of two components: “problem finding” and “problem-solving.” Problem finding refers to detecting gaps, errors, and inconsistencies in assumptions, arguments, or information, while problem-solving refers to solving problems correctly once they are identified. These different kinds of thinking were labeled as strategic reasoning and tactical reasoning.

In formulating this measure, Hogan et al. (2009) proposed that critical business reasoning involves the ability to detect covariations (i.e., identify events that go together reliably) and to recognize when a sequence is recurring or about to reoccur. At a deeper level, critical business reasoning also involves recognizing when covariations do not occur or identifying exceptions to sequences of events. When applied to a business context, critical business reasoning includes the following: (a) accurately forecasting sequences of events both within and outside one’s organization, (b) recognizing when those forecasts are applicable and when they are not, and (c) making appropriate business decisions based on those forecasts. They developed a measure to quantify individual differences in this ability—the Hogan Business Reasoning Inventory (HBRI)—which we used in this study. It is an ability measure with the possibility of deriving subscale scores such as spatial, mathematical, and verbal skills, all of which are related to success in business.

The manual of the Hogan Business Reasoning Inventory (HBRI) notes that it was created based on the fundamental need for organizations to fairly and accurately evaluate candidates’ business reasoning skills. It is based on three assumptions: *first*, that good business decisions require clear thinking, rational analysis, and critical evaluation; *second*, that business reasoning skills can be measured and that the results of this measurement process can be used to evaluate candidates for managerial and executive positions; and *third,* that the results of this measurement process predict managerial success.

### Personality and intelligence at work: executive performance

There is a vast body of literature on the relationship between personality and cognitive ability (Zeidner and Matthews, 2000). The results of many studies using different instruments and different populations, as noted above, suggest the following three viewpoints: Openness is positively associated with cognitive ability, and Conscientiousness is negatively associated with cognitive ability; the relationship is modest with small effect sizes; and the results depend largely on the measures used.

When exploring the relationship between bright-side traits and intelligence, a meta-analysis by Ackerman and Heggestad (1997) demonstrated the relationship between both higher-and lower-order personality traits (e.g., the Big Five traits and their narrower facets; Costa and McCrae, 1985) and intelligence. Test anxiety showed the strongest relationship with lower IQ scores, while Openness exhibited the strongest relationship with higher IQ scores. These findings have since been replicated (Bartels et al., 2012; Moutafi et al., 2005), confirming that high levels of Openness are the best predictor of intelligence, although the coefficients are rarely larger than 0.30. This has been shown to be true at both the domain and facet levels of the trait Openness (Furnham, 2023).

As noted above, there are specific mini-theories about the overlap between two particular traits and intelligence, which attempt to explain the often replicated results. These include compensation theory (Moutafi et al., 2004; Wood and Englert, 2009) and investment theory (von Stumm and Ackerman, 2013). There are also more recent studies that have highlighted the role of previously neglected variables such as Tolerance of Ambiguity. Indeed, in a recent study, Cuppello et al. (2023b) found that Tolerance of Ambiguity was a consistently significant positive correlate of IQ at both the facet and domain levels.

In this study, we used the Hogan Personality Inventory (HPI) to assess seven personality traits, rather than the classic Big Five traits. This results from both Extraversion and Openness being split into two traits. The seven traits are as follows: *Adjustment* (Neuroticism), *Ambition* (Leadership and Status Seeking), *Sociability* (Extraversion), *Interpersonal Sensitivity* (Agreeableness), *Prudence* (Conscientiousness), *Inquisitive* (Openness), and *Learning Approach* (Need for Intellectual Stimulation). Early research on the HPI showed that splitting Extraversion into the Ambition and Sociability components, as well as splitting Openness into the Inquisitiveness and Learning Approach components, yielded higher predictive validity in applied settings and clearer interpretation of assessment results for professional coaches. There are well over 200 published papers that have used this measure (Hogan, 2022). To the best of our knowledge, there are no studies that have linked the HPI to intelligence.

### Dark-side personality: the Hogan development survey

Nearly all studies on this topic have focused on bright-side traits and the Big Five trait correlates of different measures of Intelligence. In this study, we did the same but also examined dark-side trait correlates of business reasoning. There is a body of literature on the dark-side traits of emotional intelligence, but there are far fewer studies that have measured dark-side factors and IQ (Judge et al., 2009; Palaiou et al., 2016). However, it is possible to extrapolate from studies on the relationship between bright-and dark-side traits, as well as bight-side traits and IQ, to suggest that there may be modest correlations between Schizotypal personality disorder (PD) (Openness), OCD (Conscientiousness), and IQ. While the literature on dark-side personality traits is still developing (Kowalski et al., 2021), the idea of exploring these relationships may help move the field forward. There is a growing body of research on the connection between dark-side traits and leadership derailment or failure; however, it seems to neglect assessing the role of intelligence (Hogan et al., 2021).

An individual’s dark side is typically described within a modified taxonomy of the DSM-IV-TR manual’s classification of PDs (American Psychiatric Association, 2000). It is important to stress that dark-side traits are not, and should not be, interpreted as personality disorders for obvious ethical and legal reasons. As described by American Psychiatric Association (1994), “personality disorders must be distinguished from personality traits that do not reach the threshold for a personality disorder. Personality traits are diagnosed as a personality disorder only when they are inflexible, maladaptive, persisting, and cause significant functional impairment or subjective distress” (p. 633). The Hogan Development Survey (HDS) (Hogan and Hogan, 1997) was developed to identify non-clinical personality problems associated with career derailment and managerial failure. The 11 dimensions of the HDS are based on a taxonomy similar to that of the DSM-IV Axis II personality disorders, measuring problematic personality characteristics.

Similar to the DSM’s higher-order classification into Cluster A (Odd and Eccentric), B (Dramatic, Emotional, and Erratic), and C (Anxious and Fearful), the HDS also has three clusters: *Moving Away From Others*: People who manage insecurities by intimidating and avoiding others; *Moving Against Others*: People who expect to be liked, admired, and respected and tend to resist acknowledging their mistakes and/or failures (which they blame on others); and *Moving Toward Others*: People who want to please figures of authority.

Various studies have linked dark-side measures with work outcomes (Judge et al., 2009; Khoo and Burch, 2008; Zettler and Solga, 2013; Zibarras et al., 2008). Furnham (2006) used the HDS to explore the relationship between dark-side traits, over and above bright-side traits, and cognitive ability. When controlling for the Big Five personality traits, three dark-side traits, Skeptical, Mischievous, and Diligent, significantly predicted lower IQ across two measures of intelligence, accounting for 5% of the variance.

### The Hogan Business Reasoning Inventory

This measure evaluates critical reasoning to assess an individual’s ability to identify and solve problems. It aims to evaluate an individual’s business-related decisions using textual, graphic, and quantitative data (Akhtar et al., 2015). It describes reasoning style—the ability to evaluate sets of data, make decisions, solve problems, and avoid repeating past mistakes. By assessing reasoning style, it is possible to identify candidates’ problem-solving approach, understand their intellectual capacity, and identify areas for development. It yields two scores: *Qualitative reasoning,* which involves working with data visualization, logic, and verbal information to solve problems, and *Quantitative reasoning,* which involves working with mathematical and spatial information to solve problems. These are combined to get a total overall score, similar to *g* in the intelligence literature. The HBRI technical manual reports strong positive correlations with Raven’s APM-III (*r* = 0.65, *n* = 126), the General Aptitude Test Battery (*r* = 0.56, *n* = 106), and the Watson–Glaser CTA overall score of *r* = 0.66 (*n* = 276), suggesting solid construct validity.

### Current study

In this study, we examined the relationship between bright-side HPI and dark-side HDS personality traits and business reasoning, as measured by the HBRI. We hypothesized, based on the previous literature noted above, that Adjustment (low Neuroticism) (H1) (Hogan et al., 2008; Stanek and Ones, 2023), Inquisitiveness (Openness) (H2) (von Stumm, 2018; von Stumm and Ackerman, 2013), and Learning Approach (Openness) (H3) (Hogan et al., 2008) would be positively and significantly associated with business reasoning, while Prudence (Conscientiousness) (H4) (Moutafi et al., 2004, 2005) would be negatively associated with business reasoning.

Reviews suggest that the relationship between personality and intelligence—the two essential poles of differential psychology—is robust but weak, with significant correlations being small in magnitude and effect sizes modest. Undoubtedly, genetics explains the covariance between personality traits and reasoning/IQ.

This study is exploratory with regard to dark-side factors, which, by definition, are usually associated with maladaptive responses. Therefore, we expected all dark-side scores to be negatively associated with the HBRI, particularly Excitable (H5) (moody; intense but short-lived enthusiasm for people, projects, and things). However, we hypothesized that Imaginative (H6) (acting and thinking in creative but sometimes odd or unusual ways) would be positively associated with business reasoning (Akhtar et al., 2015).

## Methods

### Participants

In total, 2,342 (1,600 men and 742 women) working adults completed the assessments for either selection or individual development purposes. Their ages ranged from 20 to 63 years (M = 41.56, SD = 8.72); 84% were aged between 29 and 50 years. Approximately 65% of the participants indicated that their job level was at the managerial or executive level. The data were drawn from the Hogan Assessment archive, which records data typically collected in assessment centers across many, mainly English-speaking countries in America and Europe. Data collection methods ensure quality data through the assessment of issues such as time taken and erratic answers (Furnham et al., 2013, 2015).

### Measures

#### The Hogan Business Reasoning Inventory

The HBRI (Hogan et al., 2009) is a contextualized measure of business intelligence that is designed for use in corporate environments as a predictor of job performance and development. The HBRI includes 24 items; it is self-administered and measures verbal, quantitative, and analytical abilities using work-related stimuli and hypothetical situations. These abilities are divided into two categories: *Qualitative Reasoning* (working with data visualization, logic, and verbal information to solve problems) and *Quantitative Reasoning* (working with mathematical and spatial information to solve problems). These two components are averaged to create a total Business Reasoning score and normed from 0 to 100 using Hogan’s global norms. In this study, the average total Business Reasoning score was 56.855 (SD = 29.67). Although the HBRI items come in a variety of formats (e.g., reading images, charts, and data tables), one sample item reads: “A low priority is to urgency as a small budget is to _____,” with choices (a) low spending, (b) high spending, or (c) small payments. More sample items are available in the HBRI technical manual, which is available upon request from the HAS. The technical manual reports an internal consistency of 0.82, with a test–retest reliability of 0.92. A confirmatory factor analysis of the items showed evidence of a single factor (CFI = 0.90, TLI = 0.89, RMSEA = 0.03). At this stage, there is limited evidence of the discriminant validity of this test.

#### The Hogan Personality Inventory

The HPI (Hogan and Hogan, 1997) consists of 206 items that are used to produce seven personality traits and six criterion scores. Participants respond with either “True” or “False” to each item. The seven personality traits are *Adjustment* (Neuroticism), *Ambition* (Leadership and Status Seeking), *Sociability* (Extraversion), *Interpersonal Sensitivity* (Agreeableness), *Prudence* (Conscientiousness), *Inquisitive* (Openness), and *Learning Approach* (Need for Intellectual Stimulation). The scales’ internal consistency and test–retest reliabilities are well established. Both the manual and independent research have cited internal consistency alpha values above 0.71 and test–retest reliabilities ranging between 0.74 and 0.86 (Hogan and Holland, 2003; Hogan J. et al., 2007; Hogan R. et al., 2007).

#### The Hogan Development Survey

The HDS (Hogan and Hogan, 1997) quantifies behavioral syndromes that frequently impair work performance. The HDS taxonomy is similar to the classical personality disorders (PDs) described in the DSM-IV-TR. However, the HDS is not clinical-grade and cannot be used to diagnose clinical disorders. The HDS consists of 154 items, which participants complete by indicating either their agreement or disagreement. The items are scored across11 scales: *Excitable* (Borderline PD), *Skeptical* (Paranoid PD), *Cautious* (Avoidant PD), *Reserved* (Schizoid PD), *Leisurely* (Passive-Aggressive PD), *Bold* (Narcissistic PD), *Mischievous* (Antisocial PD), *Colorful* (Histrionic PD), *Imaginative* (Schizotypal PD), *Diligent* (Obsessive-Compulsive PD), and *Dutiful* (Dependent PD). The scale has been found to predict work preferences (Furnham et al., 2012) and selection effectiveness (Knights and Kennedy, 2006). The manual reports internal reliabilities ranging between 0.50 and 0.79 (average alpha = 0.67) and test–retest reliabilities between 0.58 and 0.87 (average = 0.75).^1^

### Procedure

Participants completed all three measures online as part of their organization’s requirements. Reports containing the results of these assessments were provided to the client as part of the contractual obligation. The data were anonymized for the purposes of this research. Data inspection showed no signs of erratic responses.

### Design

This was a simple correlational study, which, by virtue of the large sample size, had sufficient statistical power. The study used a convenience sample based on the test publisher’s data bank. As a result, the sample was inevitably restricted to working individuals, primarily those in middle management roles across international contexts. The proposed analysis involved first examining correlations, followed by regression analyses with the HBRI score as the dependent variable.

## Results

Table 1 shows the correlation between all three measures and the Cronbach’s alpha values. All values except two exceeded 0.60, with most being above 0.70.

**Table 1:** Alpha values of all variables and correlations between the three questionnaires.

		Alpha	1	2	3	4	5	6	7	8	9	10	11	12	13	14	15	16	17	18
(1)	Adjustment	0.83																		
(2)	Ambition	0.78	0.44^**^																	
(3)	Sociability	0.79	0.12^**^	0.33^**^																
(4)	Interpersonal sensitivity	0.61	0.48^**^	0.31^**^	0.33^**^															
(5)	Prudence	0.64	0.47^**^	0.15^**^	−0.09^**^	0.32^**^														
(6)	Inquisitive	0.75	0.19^**^	0.30^**^	0.35^**^	0.21^**^	0.05^*^													
(7)	Learning approach	0.73	0.24^**^	0.34^**^	0.18^**^	0.18^**^	0.15^**^	0.43^**^												
(8)	Excitable	0.66	−0.64^**^	−0.39^**^	−0.20^**^	−0.43^**^	−0.36^**^	−0.17^**^	−0.18^**^											
(9)	Skeptical	0.70	−0.54^**^	−0.30^**^	−0.10^**^	−0.34^**^	−0.33^**^	−0.09^**^	−0.14^**^	0.57^**^										
(10)	Cautious	0.68	−0.43^**^	−0.64^**^	−0.31^**^	−0.29^**^	−0.17^**^	−0.26^**^	−0.30^**^	0.36^**^	0.33^**^									
(11)	Reserved	0.70	−0.29^**^	−0.35^**^	−0.30^**^	−0.45^**^	−0.25^**^	−0.17^**^	−0.13^**^	0.36^**^	0.36^**^	0.35^**^								
(12)	Leisurely	0.58	−0.31^**^	−0.30^**^	−0.07^**^	−0.19^**^	−0.17^**^	−0.05^*^	−0.08^**^	0.28^**^	0.39^**^	0.33^**^	0.29^**^							
(13)	Bold	0.72	−0.03	0.15^**^	0.27^**^	0.05^*^	0.00	0.21^**^	0.22^**^	0.05^**^	0.20^**^	−0.14^**^	0.05^*^	0.25^**^						
(14)	Mischievous	0.67	−0.24^**^	0.09^**^	0.39^**^	−0.03	−0.48^**^	0.24^**^	0.06^**^	0.16^**^	0.25^**^	−0.09^**^	0.04	0.18^**^	0.30^**^					
(15)	Colorful	0.70	0.00	0.33^**^	0.63^**^	0.19^**^	−0.13^**^	0.28^**^	0.21^**^	−0.06^**^	0.02	−0.33^**^	−0.19^**^	0.05^*^	0.42^**^	0.51^**^				
(16)	Imaginative	0.69	−0.04^*^	0.21^**^	0.37^**^	0.07^**^	−0.23^**^	0.36^**^	0.24^**^	0.02	0.12^**^	−0.15^**^	0.02	0.17^**^	0.45^**^	0.52^**^	0.47^**^			
(17)	Diligent	0.63	−0.02	0.03	0.00	0.04^*^	0.26^**^	0.16^**^	0.15^**^	0.11^**^	0.12^**^	0.01	0.05^*^	0.14^**^	0.28^**^	−0.06^**^	0.02	0.11^**^		
(18)	Dutiful	0.55	0.00	−0.15^**^	0.07^**^	0.15^**^	0.20^**^	0.00	−0.04	−0.03	0.04	0.17^**^	−0.03	0.10^**^	0.06^**^	−0.07^**^	0.02	−0.04	0.21^**^	
(19)	HBRI	0.85	0.09^**^	0.14^**^	0.08^**^	0.03	−0.06^**^	0.03	0.14^**^	−0.12^**^	−0.13^**^	−0.07^**^	−0.01	−0.15^**^	−0.08^**^	−0.01	−0.01	0.04^*^	−0.13^**^	−0.09^**^

**Correlation is significant at the 0.01 level (2-tailed). *Correlation is significant at the 0.05 level (2-tailed).

To test the hypotheses, correlational analysis was conducted, followed by regression analyses.

Table 2 shows the correlations, indicating that five of the seven traits were significantly correlated with Business Reasoning (BR). The two strongest correlations were for Ambition and Inquisitive. Furthermore, the male participants scored higher than the female participants (Male participants: *M* = 58.74, *SD* = 29.21; Female participants: *M* = 51.83, *SD* = 30.13; Cohen’s *d* = 0.23). Table 3 shows the second step in the two-step regression analysis. It indicates that the following three scales were positively associated with BR: Adjustment, Ambition, and Learning Approach. In contrast, Prudence and Inquisitive were negatively associated with BR. Overall, the predictor variables accounted for just over 5% of the total variance.

**Table 2:** Means, SDs, and correlations of the BR score with the demographics and HPI factors.

	Mean	SD	BR	Age	Gender	Adjustment	Ambition	Sociability	Interpersonal sensitivity	Prudence	Inquisitive
BR	56.55	29.68									
Age	41.58	8.73	0.02								
Gender	1.32	0.47	−0.11***	−0.20***							
Adjustment	58.83	29.38	0.09***	0.06**	−0.06**						
Ambition	66.26	27.38	0.14***	0.18***	−0.15**	0.44***					
Sociability	57.18	28.12	0.08***	−0.10***	−0.02	0.12***	0.33***				
Interpersonal sensitivity	65.15	28.60	0.03	0.01	0.07**	0.48***	0.31***	0.33***			
Prudence	60.44	27.98	−0.06**	0.04	0.03	0.47***	0.16***	−0.09***	0.32***		
Inquisitive	66.72	26.34	0.03	−0.04	−0.19***	0.19***	0.30***	0.35***	0.21***	0.05*	
Learning approach	64.25	28.15	0.14***	0.06**	−0.05*	0.24***	0.34***	0.18***	0.18***	0.15***	0.43***

*p < 0.05, **p < 0.01, ***p < 0.001.

**Table 3:** Regression of the demographics and HPI bright-side traits on the BR score.

	*B*	*SE*	β	*t*
Age	−0.059	0.072	−0.017	−0 0.817
Gender	−6.479	1.357	−0.102	−4.775***
Adjustment	0.097	0.027	0.096	3.593***
Ambition	0.079	0.027	0.073	2.921**
Sociability	0.048	0.025	0.045	1.894
Interpersonal sensitivity	−0.015	0.026	−0.015	−0.587
Prudence	−0.127	0.025	−0.120	−5.061***
Inquisitive	−0.108	0.027	−0.096	−3.998***
Learning approach	0.149	0.024	0.141	6.088***
Adjusted *R*^2^		0.053		
*F*		15.520		
*p*		<0.001		

*p < 0.05, **p < 0.01, ***p < 0.001.

Table 4 shows the correlations, indicating that eight of the 11 traits were significantly correlated with BR. All correlations were negative except for Imaginative. Table 5 shows the second step in the two-step regression analysis. Among the dark-side traits, three were more significantly negatively associated with BR, namely Leisurely, Diligent, and Reserved. In contrast, Imaginative was positively associated with BR. Overall, the predictors accounted for just over 6% of the total variation in the BR scores.

**Table 4:** Means, SDs, and correlations of the BR score with the demographics and HDS factors.

	Mean	SD	BR	Age	Gender	Excitable	Skeptical	Cautious	Reserved	Leisurely	Bold	Mischievous	Colorful	Imaginative	Diligent
BR	56.55	29.68													
Age	41.58	8.73	0.02												
Gender	1.32	0.47	−0.11***	−0.12***											
Excitable	50.02	26.77	−0.12***	−0.05*	0.02										
Skeptical	51.13	27.37	−0.13***	−0.12***	0.01	0.57***									
Cautious	44.15	26.84	−0.07***	−0.12***	0.14***	0.36***	0.33***								
Reserved	49.19	28.09	−0.01	−0.13***	0.02	0.36***	0.36***	0.35***							
Leisurely	50.35	27.47	−0.15***	−0.15***	0.03	0.28***	0.40***	0.33***	0.30***						
Bold	62.86	26.81	−0.08***	−0.13**	−0.00	0.06**	0.20***	−0.15***	0.05*	0.25***					
Mischievous	54.84	28.53	−0.01	−0.03	−0.10***	0.16**	0.25***	−0.09***	0.04	0.18***	0.30***				
Colorful	57.11	26.84	−0.01	−0.03	−0.02	−0.06**	0.02	−0.33***	−0.19***	0.05*	0.42***	0.51***			
Imaginative	63.15	26.27	0.04*	−0.09***	−0.06**	0.02	0.12***	−0.15***	0.02	0.17***	0.45***	0.52***	0.47***		
Diligent	62.68	26.98	−0.13***	−0.12***	0.03	0.11***	0.12***	0.01	0.05*	0.14***	0.28***	−0.06**	0.02	0.11***	
Dutiful	58.31	27.28	–0.09***	−0.18***	0.10***	−0.03	0.04	0.17***	−0.03	0.10***	0.06**	−0.07**	0.02	–0.04	0.21***

*p < 0.05, **p < 0.01, ***p < 0.001.

**Table 5:** Regression of the demographics and HDS dark-side traits on the BR score.

	*B*	*SE*	β	*t*
Age	−0.107	0.072	−0.031	−1.485
Gender	−6.374	1.320	−0.100	−4.828***
Excitable	−0.075	0.028	−0.067	−2.633**
Skeptical	−0.063	0.029	−0.058	−2.193*
Cautious	0.012	0.028	0.011	0.444
Reserved	0.072	0.024	0.068	2.928**
Leisurely	−0.130	0.025	−0.121	−5.131***
Bold	−0.066	0.028	−0.059	−2.368*
Mischievous	−0.015	0.028	−0.014	−0.531
Colorful	−0.008	0.030	−0.007	−0.262
Imaginative	0.125	0.029	0.110	4.264***
Diligent	−0.100	0.024	−0.091	−4.149***
Dutiful	−0.056	0.023	−0.051	−2.387*
Adjusted R^2^		0.064		
*F*		13.289		
*p*		<0.001		

*p < 0.05, **p < 0.01, ***p < 0.001.

Various additional analyses were conducted to examine the incremental validity of the bright-side HPI traits over the dark-side HDS traits and vice versa. In both cases, the percentage of the variance accounted for doubled in effect, increasing from approximately 5–10%, when the second test was added. For instance, we conducted a three-step regression analysis beginning with gender and age, followed by the seven HPI traits and then the 11 HDS scales. The full model was significant, *F*(20, 2,321) = 14.08, *p*-value <0.001, and AdjR^2^ = 0.10. A total of four of the 13 scales were significant (*p* < 0.001), with the largest being HPI Ambition (*β* = 0.10, *t* = 3.67), along with HPI Sociability (*β* = 0.12, *t* = 4.32), HDS Leisurely (*β* = −0.10, *t* = 4.43), and HDS Diligent (*β* = −0.09, *t* = 3.87).

Finally, the three higher-order factors from the HDS were computed: Moving Away From Others (Cluster A), Moving Against Others (Cluster B), and Moving Toward Others (Cluster C). A regression analysis was conducted with gender and age in the first step and the three higher-order factors in the second step. The model was statistically significant, *F*(5, 2,334) = 22.09, a *p* < 0.0.001, and AdjR^2^ = 0.04. The following two clusters were negatively associated with BR: Moving Away From Others (*β* = 0.12, *t* = 5.87, *p* < 0.001) and Moving Toward Others (*β* = −0.13, *t* = 6.91, *p* < 0.001).

## Discussion

In many ways, these results align with findings from the extensive and growing literature on the intelligence-personality relationship (Bardach et al., 2024; Stanek and Ones, 2023), confirming the idea that the HBRI is a measure of “applied intelligence.” First, the relationships were modest but predictable and, by and large, replicated those found in other studies. Second, certain traits, such as Neuroticism/Adjustment and Conscientiousness/Prudence, were consistently related to many different measures of both personality and intelligence (Stanek and Ones, 2023). Third, while the relationships were significant, the association between ability and personality was modest, suggesting that these two major areas of differential psychology are distinct. However, this study contributes to the literature by examining dark-side correlates of cognitive ability in applied settings.

This study yielded some particularly interesting findings, mainly due to the novel aspects of the HPI. This measure divides Introversion-Extraversion into *Ambition,* which assesses social self-confidence, leadership potential, competitiveness, and energy and *Sociability,* which assesses the need for/enjoyment of interacting with others. Similarly, Openness is divided into two scales: *Inquisitive/Intellectance*—bright creative and an interest in intellectual matters—and *Learning Approach/Scholarship*—enjoyment of academic activities and valuing education for its own sake.

This study shows that the social confidence and energetic/competitive aspects of Extraversion, rather than the sociability factor, are relevant to business reasoning. Several studies have noted that many conceptions of the trait Extraversion contain mixed factors (Wilt and Revelle, 2008). Wolf and Ackerman (2005) published a meta-analytic investigation fifteen years ago examining the relationship between Extraversion and intelligence. They recommended splitting Extraversion into two distinct but related factors of *social potency* (broad interpersonal effectiveness and a desire to make an impact on others) and *social closeness* (warmth and need for intimacy). Overall, the relationship was weaker for social closeness compared to social potency. This indeed was the case in this study. This partially explains why studies examining the relationship between Extraversion and Leadership are inconsistent, as they tend to use different measures of Extraversion.

It was very interesting to observe the role of ambition, one of the strongest correlates of the business reasoning measure. This study replicated the study by Akhtar et al. (2015) on a much smaller population. In a recent paper titled “Hire Ambitious People,” Furnham et al. (2023) found that this trait was most closely associated with job engagement. Ambition could be seen as a trait linked to Need for Achievement, which implies that ambitious people invest efforts in improving their business reasoning and related skills. In this sense, it is also an “investment trait” and well worth assessing in potential business candidates.

Of particular interest is the unexpected relationship between Openness facets and business reasoning. Specifically, Inquisitiveness correlated negatively, while Learning Approach correlated positively, with business reasoning. Indeed, previous studies have suggested that the trait Openness correlates very differently with business outcomes such that it seems more closely related to the appreciation of creativity than the production of creative ideas. According to the HPI manual, Inquisitiveness is defined as being imaginative, quick-witted, and creative but easily bored, while Learning Approach reflects an interest in academic activities and success. This may explain why Inquisitiveness is not a marker of business reasoning, as it is more associated with “out-of-the-box” or “blue-sky” thinking—essentially divergent thinking—rather than the critical reasoning and convergent thinking typically associated with academic approaches to learning and thinking. This aligns with the literature on personality, intelligence, and creativity (Furnham, 2020) and suggests that it is possibly more important for a business leader to be able to evaluate creative ideas than to produce them. Therefore, the ability to evaluate the usefulness and practicality of a new idea, product, or process may be unrelated or indeed negatively related compared to the ability to produce those ideas.

A second novel feature of this study is the examination of dark-side correlates of business reasoning (Burch and Foo, 2010). The results revealed a number of negative associations, particularly with the traits of Leisurely, Diligent, and Excitable. Interestingly, two factors were positively associated with BR: Reserved and Imaginative. Reserved is defined as *aloof, detached, and uncommunicative; lacking interest in or awareness of the feelings of others,* while Imaginative is defined as *acting and thinking in creative and sometimes odd or unusual ways*. It is possible that Reserved people are those typical cerebral academic types with low EQ who thrive in certain parts of certain organizations (accounting, engineering) and would enjoy and perform well on business reasoning tests.

It is more difficult to explain the association with Imaginative, given that it is similar to HPI Inquisitiveness (*r* = 0.38, *p* < 0.001), which was negatively correlated with BR. It seems that Inquisitiveness reflects more curiosity and distractibility, which are unrelated to intellect. Imaginative, while also related to creativity, seems to have a deep-thinking component, which is why it was positively correlated with the business reasoning scores. Imaginative people tend to think deeply about problems for long periods of time. Individuals who are high on Inquisitiveness tend to think about all sorts of things but not for very long. However, this result requires replication and a clearer analysis of the underlying cause of the relationship.

It is also interesting to note that sub-clinical boldness (Narcissism) was negatively associated with cognitive ability, which may partly explain why, as a dark-side trait, it is associated with leadership emergence but not effectiveness. It may well be that narcissists believe they are more intelligent than they actually are and convince others of the same (Furnham et al., 2012). Indeed, it is one of the most consistent and powerful markers of leadership derailment and business failure (Hogan et al., 2021).

Another result worth noting is the gender difference. The debate over gender differences in both intelligence and personality remains contentious and highly sensitive (Eagly and Revelle, 2022; Furnham and Grover, 2022; Furnham and Treglown, 2021). The gender differences found in this study align with those established elsewhere and are essentially related to agency and communion. The thing of interest and concern is the difference in the BR scores, which showed that the male participants scored higher than the female participants, albeit with a modest effect size (*d* = 0.23). In this study, there were more than twice as many men as women (1,600 vs. 742) and the men were significantly older than the women by over 3 years. Therefore, these may not be equivalent samples. However, given the potential for adverse impact against women in selection or promotion decisions based on these data, this issue warrants further exploration. Indeed, in the interests of diversity and inclusion, it is important to explore a range of demographic differences in the HBRI to ensure that fair procedures are followed.

## Limitations and recommendations

The results suggest that the HBRI may be a useful, brief, and highly face-valid measure for assessing managerial potential, although further validation is required. This measure requires further analysis. Despite having a large sample size, we lacked information about the participants’ educational background, work history, their success at work, and how their colleagues rated them. Moreover, the reliance on self-report data introduced inherent limitations.

We recommend that future studies should gather data on the convergent and divergent validity of the HBRI to clarify its association with crystallized and fluid intelligence, as well as with other tests designed to measure cognitive ability in work settings, such as the Watson–Glaser test (Watson and Glaser, 1980).

Moreover, it is essential to understand how, when, and why business reasoning is related to business success or failure. However, it is particularly difficult to obtain data for this type of analysis, as all I/O and work psychologists are well aware.

## Data Availability

The data analyzed in this study is subject to the following licenses/restrictions: It is confidential and owned by the consultancy. Requests to access these datasets should be directed to contact the second author.
